# Meningiomas of the rolandic region: risk factors for motor deficit and role of intra-operative monitoring

**DOI:** 10.1007/s00701-023-05630-6

**Published:** 2023-06-05

**Authors:** Francesco Maiuri, Sergio Corvino

**Affiliations:** Department of Neuroscience and Reproductive and Odontostomatological Sciences, Neurosurgical Clinic, University of Naples “Federico II”, School of Medicine, Via Pansini, 5, 80131 Naples, Italy

**Keywords:** Meningioma, Rolandic region, Parasagittal, Convexity, Falx, Intraoperative monitoring

## Abstract

**Objective:**

Meningiomas of the rolandic region are associated to high risk of postoperative motor deficits. This study discusses the factors affecting motor outcome and recurrences from the analysis of a monoinstitutional case series and eight studies from a literature review.

**Methods:**

Data of 75 patients who underwent surgery for meningioma of the rolandic region were retrospectively reviewed. The analyzed factors included tumor location and size, clinical presentation, magnetic resonance imaging (MRI) and surgical findings, brain-tumor interface, extent of resection, postoperative outcome and recurrence. Eight studies from literature on rolandic meningiomas treated with or without intraoperative monitoring (IOM) were reviewed with the aim to define the impact of IOM on the extent of resection and motor outcome.

**Results:**

Among the 75 patients of the personal series, the meningioma was on the brain convexity in 34 (46%), at the parasagittal region in 28 (37%) and at the falx in 13 (17%). The brain-tumor interface was preserved in 53 cases (71%) at MRI and in 56 (75%) at surgical exploration. Simpson grade I resection was obtained in 43% of patients, grade II in 33%, grade III in 15% and grade IV in 9%. The motor function worsened postoperatively in 9 among 32 cases with preoperative deficit (28%) and in 5 among 43 with no preoperative deficit (11.5%); definitive motor deficit was evidenced in overall series at follow-up in 7 (9.3%). Patients with meningioma with lost arachnoid interface had significant higher rates of worsened postoperative motor deficit (*p* = 0.01) and seizures (*p* = 0.033). Recurrence occurred in 8 patients (11%). The analysis of the 8 reviewed studies (4 with and 4 without IOM) shows in the group without IOM higher rates of Simpson grades I and II resection (*p* = 0.02) and lower rates of grades IV resection (*p* = 0.002); no significant differences in postoperative immediate and long-term motor deficits were evidenced between the two groups.

**Conclusions:**

Data from literature review show that the use of IOM does not affect the postoperative motor deficit Therefore, its role in rolandic meningiomas resection remains to be determined and will be defined in further studies.

## Introduction

Surgery of rolandic meningiomas is challenging because of their close anatomical relationship to the highly functional underlying area, represented by the sensory-motor cortex [29–31]. Optimal surgical management should consider an onco-functional balance, striving for maximal safe resection while preserving the function and improving/restoring the clinical symptoms [10]. In this setting while choosing an aggressive surgical approach to attempt a gross total resection might lead to unnecessary peri- and postoperative morbidity, a less invasive and more conservative treatment whit the goal of a subtotal resection followed by adjuvant treatments might not lead to clinical symptoms improvement/restoration and increase the recurrence rate [21, 22, 24].

Several Magnetic Resonance Imaging (MRI) and surgical findings are related to the risk of postoperative neurological worsening, such as the absence of a well-defined brain-tumor interface resulting in high adherence between tumor and motor cortex, the presence of irregular tumor margins [3, 31], the close adherence to the bridging veins, the invasion of the superior sagittal sinus. Therefore, the best management of these surgical problems, i.e. more versus less aggressive approach, is still controversial.

This study reports a monoinstitutional series of 75 meningiomas of the rolandic region and reviews 8 other studies from the literature of patients treated with or without intraoperative neurophysiological monitoring (IOM) [3, 4, 7, 17, 29–31, 35] in order to discuss the optimal surgical management to decrease the risk of postoperative motor deficits.

## Materials and methods

### Patient population

Data of seventy-five patients with histological diagnosis of meningioma localized to the rolandic region operated on at the Neurosurgical Clinic of the University “Federico II”, School of Medicine of Naples between 1990 and 2015 were reviewed.

Inclusion criteria were localization at the rolandic region, cases with complete clinical, MRI and surgical data, WHO histological grades I and II, follow-up > 5 years. All tumors were classified according to the 2016 WHO Classification of tumors of the central nervous system.

Cases with incomplete data, multiple and malignant (WHO grade III) meningiomas were excluded.

### Analyzed factors

Patients’ medical history, MRI studies, surgical descriptions and follow-up data of the patients were reviewed. The analyzed factors included symptoms and signs at diagnosis, tumor location and size, tumor margins, brain-tumor interface, involvement of the superior sagittal sinus, extent of resection, WHO grade, postoperative neurological complications and recurrences.

According to the location, meningiomas were classified in convexity, parasagittal and falcine. The tumor location at the rolandic region was verified by using the classic anatomical landmarks on the preoperative contrast-enhanced magnetic resonance imaging scans, including the coronal suture, the precentral and central sulci, and the rolandic vein [6].

MRI findings included tumor margins, tumor size, brain-tumor interface, involvement of the superior sagittal sinus. Tumor margins were classified into smooth and irregular. Tumor size was defined by maximum diameter and was classified into 3 groups: group 1 ≤ 3 cm, group 2 between 3–5 cm and group 3 > 5 cm.

The involvement of the superior sagittal sinus was evaluated in parasagittal meningiomas and was classified into three groups: type 1: normal or narrowed caliber < 50%; type 2: narrowed > 50%; type 3: occluded.

Descriptions of the surgical procedure were reviewed to evaluate the brain-tumor interface, which was defined as preserved or lost, and the involvement of the superior sagittal sinus. The extent of surgical resection was defined on the basis of both intraoperative finding and postoperative post-contrast MRI studies according to the Simpson grading system [33].

The WHO grade was defined according to the WHO 2016 classification [18].

The follow-up ranges from 7 to 29 years.

The recurrence rate was defined based on follow-up MRI studies. “Recurrence” was defined as evidence of a new intradural tumor after Simpson grades I and II resections, unlike the “progression” which was defined as increase in size of residual tumor after Simpson grades III and IV resections.

### Literature review

A Medline search was made from 1990 to 2023 in PubMed and Embase online electronic databases by providing the following words: “meningioma”, “rolandic region”, “convexity”, “parasagittal”, “falx”. Inclusion criteria were surgical series, reviews and case reports in English language, as well as papers written in other languages but including the abstract in English, and reporting meningioma location in the rolandic region, extent of surgical resection according to the Simpson [33] grade, preoperative motor deficits, outcome of the motor function. Studies on meningiomas of all locations without separate data on rolandic ones, studies including both intracerebral and extracerebral rolandic tumors with no data on meningiomas and series with incomplete data were excluded. From this review only 8 studies were considered eligible for the analysis [3, 4, 7, 17, 29–31, 35] (Graphic 1).

**Graphic 1:**
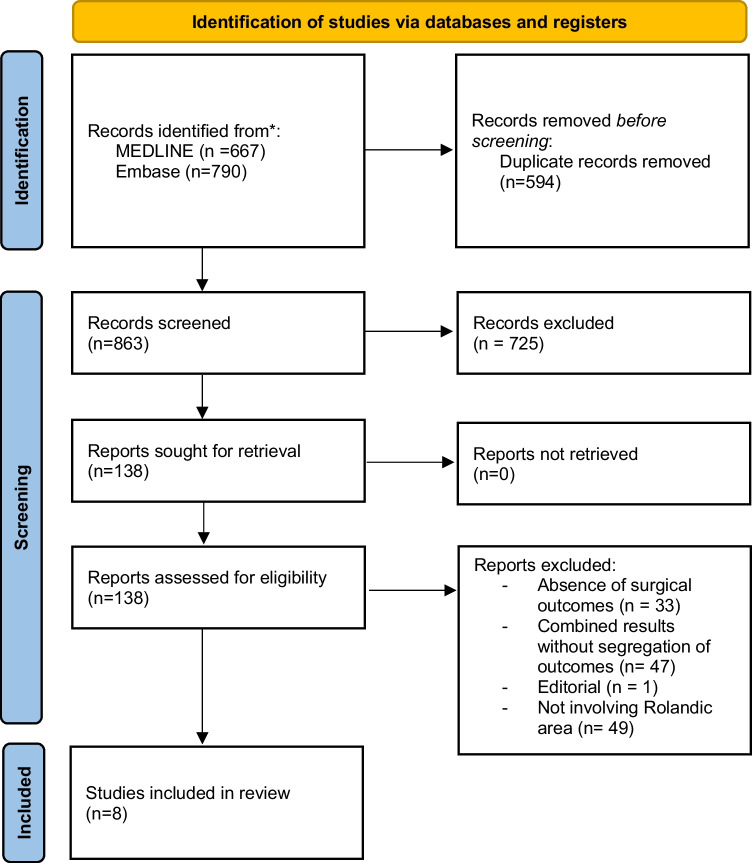
Flow chart showing the methods for the selection of the studies included in the review. *Consider, if feasible to do so, reporting the number of records identified from each database or register searched (rather than the total number across all databases/registers). **If automation tools were used, indicate how many records were excluded by a human and how many were excluded by automation tools. *From:* Page MJ, McKenzie JE, Bossuyt PM, Boutron I, Hoffmann TC, Mulrow CD, et al. The PRISMA 2020 statement: an updated guideline for reporting systematic reviews. BMJ 2021;372:n71. https://doi.org/10.1136/bmj.n71. For more information, visit: http://www.prisma-statement.org/

Based on the use of IOM, the studies were grouped as follows: group A (4 studies) including patients operated on with microsurgical technique and without IOM [3, 7, 17, 30]; group B (4 studies) including patients operated on with IOM [4, 29, 31, 35]. In three of the group B studies the IOM was routinely used [4, 29, 35], while in only one [31] the navigated transcranial magnetic stimulation (nTMS) was first used to identify the cortical motor area and the IOM was mainly used in cases with loss of the arachnoid interface.

Meningioma location, Simpson grade of surgical resection, preoperative motor deficits and outcome of the motor function were analyzed in both groups and statistically compared.

### Statistical analysis

Fisher Exact tests were used for individual variables. *P* values smaller than 0.05 were considered significant.

## Results

### Patient features, tumor location and pathology

The 75 patients were 48 females and 27 males ranging in age from 28 to 76 years old (median 53 years). Tumor location was the brain convexity in 34 cases (46%), the parasagittal region in 28 (37%) and the falx in 13 (17%) (Table 1). The tumor was on the right side in 35 patients (47%) and on the left side in 40 (53%). Seventy patients presented with neurological symptoms including headache in 30 (40%), focal or generalized sensory-motor seizures in 36 (48%) and arm and/or leg motor deficits in 32 (43%). Five patients (7%) with no or unrelated symptoms were operated on because of the large tumor size.

**Table 1:** Demographic, clinical and pathological features

Covariates	Number of patients 75 (100%)
Sex	
- F	48 (64%)
- M	27 (36%)
Age range	28–76 y (median 53y)
Meningioma location	
- Convexity	34 (46%)
- Parasagittal	28 (37%)
- Falx	13 (17%)
Side	
- Right	35 (47%)
- Left	40 (53%)
Sign and symptoms at diagnosis	
- Headache	30 (40%)
- Seizures	36 (48%)
- Motor deficits	32 (43%)
- Asymptomatic	5 (7%)
WHO grade	
- I	63 (84%)
- II	12 (16%)

According to the 2016 WHO Classification [18], 63 meningiomas (84%) were grade I and 12 (16%) were grade II.

All these data are summarized in Table 1.

### MRI findings

**Fig. 1:**
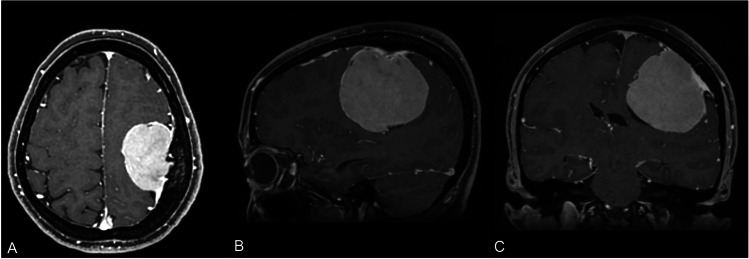
Contrast-enhanced brain MRI, axial (A), sagittal (B) and coronal (C) TI-weighted sequences: large (6 cm) left rolandic meningioma with radiologic findings of benign (WHO grade I) histotype

**Fig. 2:**
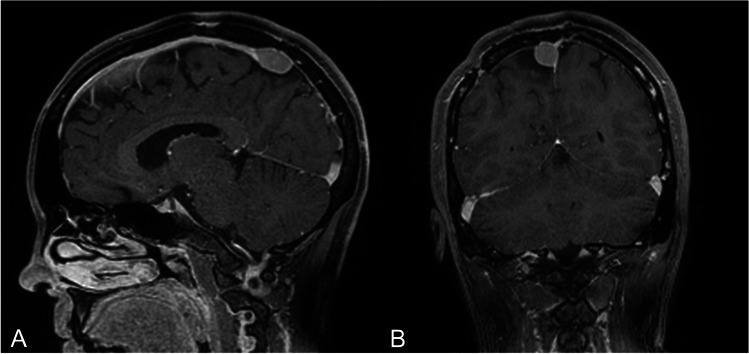
Contrast-enhanced brain MRI, sagittal (A) and coronal (B) TI-weighted sequences: recurrence of a right parasagittal rolandic meningioma treated by Simpson grade III resection with narrowed superior sagittal sinus > 50%

Tumor size was < 3 cm in 18 cases (24%), between 3 and 5 cm in 46 (61%) and > 5 cm in 11 (15%). Tumor margins were smooth and regular in 56 cases (75%) and irregular in 19 (25%). The brain-tumor interface was considered as preserved in 53 patients (71%) and lost in 22 (29%) (Fig. 1). Among the 28 parasagittal meningiomas, the superior sagittal sinus was normal or narrowed < 50% in 14 patients (50%), narrowed > 50% in 9 (32%) and completely occluded in 5 (18%) (Fig. 2). All these data are summarized in Table 2.

**Table 2:** MRI findings at initial diagnosis

Covariates	Number of patients 75 (100%)
Tumor size (cm)	
- < 3 cm	18 (24%)
- 3–5 cm	46 (61%)
- > 5 cm	11 (15%)
Tumor margins	
- Smooth	56 (75%)
- Irregular	19 (25%)
Brain-tumor interface	
- Preserved	53 (71%)
- Lost	22 (29%)
Superior sagittal sinus caliber (28 parasagittal meningiomas)	
- Normal or narrowed < 50%	14 (50%)
- Narrowed > 50%	9 (32%)
- Complete occlusion	5 (18%)

### Surgical findings

At the microsurgical exploration, the arachnoid interface was preserved in 56 cases (75%), where a clear extra-pial surgical plane was found. In 19 patients (25%) the arachnoid interface was lost in many points and resection was made through a subpial surgical plane or leaving a very small tumor fragment in most adherent points. In the 28 parasagittal meningiomas, the intradural tumor was resected and the sinus wall was coagulated.

The extent of resection was Simpson grade I in 32 patients (43%), grade II in 25 (33%), grade III in 11 (15%) and grade 4 in 7 (9%). All these data are summarized in Table 3.

**Table 3:** Surgical findings

Covariates	Number of patients 75 (100%)	Location		
Arachnoid interface				
- Preserved	56 (75%)			
- Lost	19 (25%)			
Simpson grades of resection		Convexity (34)	Parasagittal (28)	Falx (13)
- I	32 (43%)	29 (85%)	–-	3 (23%)
- II	25 (33%)	1 (3%)	15 (53%)	9 (70%)
- III	11 (15)	–-	11 (40%)	–-
- IV	7 (9%)	4 (12%)	2 (7%)	1 (7%)

### Outcome

Postoperative complications consisted in hemorrhage of the surgical field in 2 cases (2.5%) (requiring reoperation in one) and wound infection in another (1.3%).

The neurological outcome was as follows (Table 4). The motor function worsened in the postoperative course in 9 among 32 patients (28%) with preoperative deficit; persistent motor deficit was observed at the follow-up in 4 (12.5%). Three other patients experienced new-onset motor deficit, for an overall rate of 7 out of 75 patients (9.3%). Seizures worsened in the postoperative period in 6 among 36 patients (17%) with preoperative epilepsy; new-onset seizures were observed in 3 among 39 patients (8%). In the overall series episodic, seizures at follow-up despite antiepileptic therapy were present in 6 patients (8%). Patients with loss of the brain-tumor interface at surgery had a significantly higher rate of persistent seizures (*p* = 0.033) and motor deficits (*p* = 0.01) (Table 5).

**Table 4:** Postoperative clinical outcome

Covariates	Preoperative		Postoperative		Worsened at follow-up
			Improved/Stable	Worsened	
Seizures	Seizures	36	30 (83%)	6 (17%)	3 (8%)
	No seizures	39	34 (87%)	5 (13%)	3 (8%)
	Total	75	64 (85%)	11 (15%)	6 (8%)
Motor deficit	Deficit	32	23 (72%)	9 (28%)	4 (12.5%)
	No deficit	43	38 (88.5%)	5 (11.5%)	3 (7%)
	Total	75	61 (81.5%)	14 (18.5%)	7 (9.3%)

**Table 5:** Correlation between neurological outcome and brain-tumor interface

Seizures and motor deficit at follow-up		Brain-tumor interface (75)		Statistical analysis *P* value
		Preserved (56)	Lost (19)	
Seizures	6	2 (3.5%)	4 (21%)	*p* = 0.033
Motor deficits	7	2 (3.5%)	5 (26%)	*p* = 0.01

Fisher exact test has been used for statistical analysis

Fourteen patients treated by grade III and IV resection underwent postoperative stereotactic radiosurgery.

Recurrences occurred in 8 cases (11%) (Table 6). The progression-recurrence rate was higher in parasagittal (18%) meningiomas than in convexity (6%) and falx (8%) meningiomas in WHO grade II (25%) versus grade I tumors (8%), and in cases with lost (16%) versus preserved (9%) arachnoid interface, but with no statistical significance. Patients who underwent Simpson grades III and IV resection showed significantly higher progression rates (27% and 28%) than those who underwent grades I (3%) or II resection (8%) (*p* = 0.016). The difference was significant for grades I-II versus III (*p* = 0.049) and not significant for grades I-II versus IV (*p* = 0.088) (Table 6).

**Table 6:** Correlation between recurrences, location, arachnoid interface, extent of resection and histological grade of meningiomas

Covariates	Meningioma location			Arachnoid interface		WHO grade		Extent of resection (Simpson grade)			
	Convexity	Parasagittal	Falx	Preserved	Lost	I	II	I	II	III	IV
Recurrence (8)	2 (6%)	5 (18%)	1 (8%)	5 (9%)	3 (16%)	5 (8%)	3 (25%)	1 (3%)	2 (8%)	3 (27%)	2 (28%)
No recurrence (67)	32 (94%)	23 (82%)	12 (92%)	51 (91%)	16 (84%)	58 (92%)	9 (75%)	31 (97%)	23 (92%)	8 (73%)	5 (72%)
Total (75)	34	28	13	56	19	63	12	32	25	11	7
Statistical analysis	*p* = 0.29			*p* = 0.4		*p* = 0.11		I vs II vs III vs IV: *p* = 0.54 I-II vs III: *p* = 0.049 I-II vs IV: *p* = 0.088 I-II vs III-IV: *p* = 0.016			

Fisher exact test has been used for statistical analysis

### Data of literature review

Data from the two groups of studies, for an overall number of 283 patients are summarized in Table 7. The two groups, without and with IOM, included a rather similar overall number of patients (145 versus 138) and rather similar rates of meningioma location at the brain convexity (59% vs 55%, *p* = 0.54), parasagittal region (26% vs 30%, *p* = 0.59) and falx (15% for both, *p* = 1). Thus, they were homogeneous in number of patients and location. The rate of complete resections (Simpson grade I and II) in group A ranged from 77 to 100% (median value of the overall group 89%). Also, in group B it ranged from 74 to 100% in 3 studies, while another reports a rate of 36.5% (median value 79%). The difference between the two groups was significant (*p* = 0.002). The rate of Simpson grade IV resection was higher in group B studies (6.5%—47.5%, median value 12%) than in group A studies (1.2%—23%, median 3%) (*p* = 0.002).

**Table 7:** Data of Rolandic meningiomas from literature review

Authors /year	N. of Cases	Location			Tumor Resection (Simpson grade)			Preoperative Motor deficit	Postoperative outcome		Clinical Worsening at follow-up	Recurrence
		Convexity	Parasagittal	Falx	I-II	III	IV		Worsening—stable	Motor deterioration		
Group A—(No IOM)												
Bi et al. [3]. 2013	26	–-	26 (100%)	–-	20 (77%)	6 (23%)	–-	16 (61%)	17 (65%)	9 (35%)	2 (4mo.) (7.7%)	None (average 20mo.)
Lee et al. [17]. 2016	13	13 (100%)	–-	–-	10 (77%)	–-	3 (23%)	5 (39%)	13 (100%)	–-	–-	n.s
Elzarief et al. [7]. 2018	17	–-	12 (71%)	5 (29%)	17 (100%)	–-	–-	8 (47%)	8 (47%)	9 (53%)	3 (18%)	n.s
Ottenhausen et al. [30]. 2018	89	72 (81%)	–-	17 (19%)	82 (92.1%)	6 (6.7%)	1 (1.2%)	53 (60%)	67 (73.5%)	22 (24.7%)	n.s	n.s
Total	145	85 (59%)	38 (26%)	22 (15%)	129 (89%)	12 (8%)	4 (3%)	82 (57%)	105 (72%)	40 (28%)	5 (9%)	
Group B—(IOM)												
Ostry et al. [29]. 2012	42	25 (59.5%)	10 (23.5%)	7 (16.7%)	31 (74%)	–-	11 (26%)	16 (38%)	34 (81%)	8 (19%)	3 (7.1%)	5 (12%)
Tang et al. [35]. 2018	30	18 (60%)	6 (20%)	6 (20%)	30 (100%)	–-	–-	14 (47%)	16 (53%)	14 (47%)	1 (3.3%)	n.s
Raffa et al. [31]. 2019	47	24 (51%)	16 (34%)	7 (15%)	41 (87%)	3 (6.5%)	3 (6.5%)	20 (42%)	34 (72%)	13 (28%)	4 (3mo.) (8.5%)	n.s
Coșman et al. [4]. 2020	19	9 (47.5%)	9 (47.5%)	1 (5%)	7 (36.5%)	9 (47.5%)	3 (47.5%)	5 (26%)	16 (84%)	3 (16%)	1 (12mo.) (5%)	n.s
Total	138	76 (55%)	41 (30%)	21 (15%)	109 (79%)	12 (9%)	17 (12%)	55 (40%)	100 (72%)	38 (28%)	9 (7%)	
Statistical significance		*P* = 0.54	*P* = 0.51	*P* = 0.99	*P* = 0.02	*P* = 0.3	*P* = 0.002	*P* = 0.08	*P* = 1	*P* = 1	*P* = 0.55	

*IOM* Intra-operative monitoring; *n.s.* Not specified

Fisher exact test has been used for statistical analysis

Preoperative motor defects were present in 57% of group A patients, compared to 40% of group B. The analysis of the outcome of the motor function after surgery (Table 7) showed no significant differences between cases with improve or stable motor function (72% of group A versus 72% of group B, *p* = 1) and cases with immediate postoperative worsening (28% in both groups, *p* = 1). The rates of permanent motor deficits at the follow-up ranged from 0 to 18% in 3 studies of group A with available data (median 9%) versus 3.3% to 8.5% (median 6.5%) of group B, with no significant difference (*p* = 0.55).

## Discussion

Although intracerebral tumors of critical regions, especially gliomas, are the object of many studies in the world literature, the problem of meningiomas of the rolandic region is less debated. Most studies[1, 9, 13–15, 19, 26, 27, 32, 34] include all meningiomas of the brain convexity, parasagittal region and falx without focusing on the rolandic location; others [8, 11, 16, 20] include tumors of the rolandic region of different origin (both intracerebral and extracerebral) without providing separate data on meningiomas.

The present study reviews the only 8 reported studies on meningiomas of the rolandic region with the aim to define the impact of the IOM in the extent of surgical resection and neurological outcome. No other studies have discussed this issue. Three out of the four studies including patients operated on with IOM have recently been published between 2018 and 2020. Our retrospective surgical series includes patients operated on before 2015 without IOM. It shows complete resection (Simpson grades I and II) in 57 (76%) patients and grade IV resection in 7 (9%). Variable worsening of the motor function persistent at the follow-up occurred in seven patients (9.3%); it was significantly correlated with the lost brain-tumor interface (*p* = 0.01).

This literature review has several limitations, including the small number of patients in some series (four with less than 30 cases), the lack of data on the brain-tumor interface (provided in only one study [29]) and the lack of grading of the preoperative and postoperative motor deficit. However, the data are surprising, because they suggest that the use of IOM results in less radical tumor resection and no significant difference in immediate and long-term worsening of the motor function.

In some reviewed studies, risk factors for postoperative deterioration of the motor function include minor (versus severe) preoperative motor deficit [31], perilesional edema, and intraoperative cleavage plane [3, 29].

The brain-tumor arachnoid interface is the most relevant factor and deserves to be discussed. Cases with loss of the arachnoid cleavage plane include two different conditions: tumor-pial adhesion and cortical invasion. According to several anatomic studies the initial segment of pyramidal cells is located approximately 1,3 mm below the cortical surface [29]; therefore, the pial adhesion often does not correspond to cortical invasion and motor cells involvement. The microsurgical management of the brain-tumor interface is very important. The tumor mass must be reduced as much as possible up to obtain a very thin tumor layer in front of the cortex. The brain-tumor interface must be carefully investigated in each point of the tumor surface. If gentle dissection is easy, the resection can be accomplished. At points where the adherence is harder, a further reduction of the residual tumor up to 1–2 mm is advisable. The bipolar coagulation at the brain-tumor interface should be avoided because it can cause vascular injury to the brain cortex. The IOM of the cortical motor area is undoubtedly useful in deciding the management of the more adherent tumor fragments (subpial resection versus maximal reduction). However, in our review the rate of Simpson grade IV resections is higher in the group of studies including cases operated on with IOM (median 12% versus 3%). This may be explained by the more prudent tumor resection on the cortical surface, which may sometimes be stopped early as result of the intraoperative stimulation.

The reasons why IOM does not protect against postoperative motor deficit deserve to be discussed. It may be suggested that the direct cortical damage is not the main mechanism. Other factors are more frequently involved, including damage of small cortical veins, minor local ischemia, local edema. IOM does not protect against these anatomical changes. Early postoperative MRI studies could evidence the extent of ischemia and edema, but they are rarely used in meningioma patients. Besides, when the motor cortex is displaced by the meningioma and is located below the lesion, it is inaccessible to the stimulation [31].

The nTMS is a useful preoperative tool which was introduced for the surgical planning of brain tumor involving eloquent areas; however, only one study [31] reports its usefulness in surgery of rolandic meningiomas. This technique allows well localizing the motor cortex and predicting the lack of arachnoid cleavage plane, thus selecting the cases which need for IOM. We think this is an important tool for surgery of meningiomas of cortical areas. The role of the preoperative motor deficit on the outcome of the motor function after surgery is suggested in one study [30]; it shows that patients with minor preoperative weakness are more likely to have a worsening of motor function than those with more significant weakness. Even large rolandic meningiomas with well-preserved brain-tumor interface may have no or moderate motor deficit due to brain compression; in such cases remission or significant motor improvement occur after microsurgical tumor resection. On the other hand, a minor preoperative deficit due to pial adhesion and/or initial cortical invasion is more likely to get worse postoperatively due to further cortical damage. Thus, in those patients a more prudent IOM assisted resection is advisable.

An unexpected worsening of the motor function may sometimes be due to a damage of the supplementary motor area [2, 28]. Although this event is more frequent in low-grade gliomas, it may also be observed for rolandic parasagittal meningiomas, which tend to dissociate the white matter fibers.

The preservation of the rolandic vein is one of the key-points of surgery of rolandic meningiomas. The vein involvement, mainly for parasagittal and parafalcine meningiomas, is an important predictive factor of postoperative worsening of the motor function and is associated to higher complication rate [5, 34]. In such patients we suggest a more balanced approach, as for those with involvement of the highly functional segments of the venous sinuses [23, 25].

The recurrence rate was cited in only 2 of the reviewed studies [3, 29]. Bi et al. [3] did not find recurrences in 21 patients with rolandic parasagittal meningiomas treated without IOM during the follow-up period ranging from 8 months to 5 years (average 20 months). Ostry et al. [29] report recurrence occurrence in 5 among 42 patients (12%) with rolandic meningiomas of all locations operated on with IOM; the recurrence rate was higher after Simpson grade IV (36.4%) than Simpson grades I and II resections (3.2%) and for WHO grade II (21.4%) versus grade I tumors (7.1%). Data of our series of patients treated without IOM suggest that the recurrence rate is mainly related to the intra-sinus residual tumor (Simpson grade III) than to the cortical tumor residual (Simpson grade IV). Thus, the role of IOM in reducing the recurrence rates of the rolandic meningiomas must be defined in further studies.

The issue of surgery of rolandic meningiomas is whether the IOM is needed and must be used routinely or in selected patients at higher risk of postoperative motor deficit, including those with loss of brain-tumor interface and perilesional edema at MRI [12, 30], and those who already present mild to moderate motor deficit. Many patients may be operated on only by microsurgical technique and shorter surgical time. Our literature review shows no improvement on extent of resection and postoperative motor outcome in surgical series of cases treated with IOM, thus suggesting that it is not needed. However, only larger series of patients will better define this question.

## Limits of the study

First limit is the retrospective nature of the study. Due to the long recruitment period (25 years), we must consider the evolution in surgical experience and the refinements of the diagnostic imaging. The literature review includes studies with small size samples and the overall number of cases is limited. Information about the arachnoid plane are lacking in all studies and the recurrence rate is reported in only two.

## Conclusion

Meningiomas of the rolandic region with lost arachnoid plane are associated to higher risk of postoperative worsening of the motor function and patients should be counselled accordingly. This review shows that IOM use does not reduce the rate of postoperative motor deficit. However, its role in the resection of rolandic meningiomas remains to be determined and will be defined by further studies including a larger number of patients.

## Data Availability

Data of the current original research are available from the corresponding author on reasonable request.

## Abbreviations

MRI: Magnetic resonance imaging
WHO: World Health Organization
IOM: Intra-operative monitoring
nTMS: Navigated transcranial magnetic stimulation
